# Does collaborative remembering serve a directive function? Examining the influence of collaborative remembering on subsequent decision making

**DOI:** 10.1177/17470218251325246

**Published:** 2025-02-27

**Authors:** Magdalena Abel

**Affiliations:** Department of Experimental Psychology, Regensburg University, Regensburg, Germany

**Keywords:** Social contagion, collaboration, false memory, decision making

## Abstract

Remembering together with others can facilitate memory for previously encountered contents, but can also prompt social contagion with information not previously encountered. This study examined whether these effects of collaborative remembering might serve a directive function and guide subsequent individual decisions. Participants were tested in groups of three and completed an adapted version of a prisoner’s dilemma. They initially encountered faces of different players on a screen, who cooperated with them or acted as cheaters. Some of these players were encountered by all three participants, others by single participants only. An interpolated memory test on all players was completed individually or collaboratively. During a final decision game, participants were asked to decide whether to cooperate with each player or not. Three experiments were conducted, which additionally varied encoding, the retention interval before the interpolated memory test, and format and instructions for the interpolated memory test. The results consistently showed adaptive decision making. Participants were more likely to cooperate with players who had previously cooperated with them, relative to both new players and cheaters. Interpolated collaborative remembering had no benefit, however—neither for decisions toward directly encountered players nor for decisions toward players encountered by other participants. Effects of collaborative remembering may thus not serve a directive function and guide future behavior, or at least they may not do so in this adapted version of a prisoner’s dilemma.

## Introduction

### Effects of collaborative remembering and their potential functions

When individuals who possess knowledge of different parts of information come together and jointly try to remember the information, this can greatly affect and change their subsequent memories. On the one hand, collaborative remembering can result in strengthening of overlapping contents that were already known by all or several group members. This effect is called collaborative facilitation, with collaboration enhancing the individuals’ memory of previously encountered contents (e.g., Abel & Bäuml, 2017; Bärthel et al., 2017; Basden et al., 2000; Blumen & Stern, 2011; Blumen et al., 2014; Weldon & Bellinger, 1997). On the other hand, collaborative remembering can also result in the spread of information that was initially only known by single group members. This effect is called social contagion, with collaboration providing an opportunity for information transmission from single individuals to others (Roediger et al., 2001; Wright et al., 2000; see also Andrews & Rapp, 2014; Andrews-Todd et al., 2021; Maswood & Rajaram, 2019). Importantly, collaborative facilitation and social contagion can arise simultaneously, when information is initially only partly shared across all group members (Abel & Bäuml, 2020, 2023).

Social contagion is usually framed negatively, as showing that individual memory can be contaminated and distorted on the basis of input from social sources. This view is highly appropriate in certain applied fields, such as eyewitness memory (e.g., Gabbert et al., 2003, 2004; Paterson et al., 2011). More generally, false memories have, however, been conceptualized as by-products of a flexible memory system, which may potentially also fulfill adaptive functions (Newman & Lindsay, 2009; see also Howe, 2011; Schacter et al., 2011). For example, it has been shown that associative false memories as examined in the DRM paradigm (Deese, 1995; Roediger & McDermott, 1995) can support subsequent problem solving (Howe et al., 2011, 2015; Thakral et al., 2023). Conceptualizing false memories in this manner connects well with an adaptive memory framework, assuming that memory has evolved to solve (fitness-relevant) problems (e.g., Nairne, 2010; Nairne & Pandeirada, 2016), and that its purpose may not be to accurately relive the past, but to address challenges in the present and future (e.g., Klein, 2013). Moreover, it also connects to the autobiographical memory literature, which stresses that memories are often used for particular purposes and fulfill a set of different functions (e.g., an identity function, a social function, and a directive function; see Bluck et al, 2005).

Social contagion has previously been suggested to serve a social function (Abel & Bäuml, 2023; see also Choi et al., 2017), such that false memories on the basis of input from social sources may help to connect us to others around us. Integrating information from other group members into one’s own memory contributes to the development of shared memories at the group level, with all group members coming to remember larger proportions of the same information. As such, social contagion and the flexibility of our memory system may be helpful for reaching a shared mnemonic basis across individuals. Interestingly, collaborative facilitation has been suggested to contribute to the development of shared memories as well (e.g., Barber et al., 2012; Blumen & Rajaram, 2008; Congleton & Rajaram, 2014; Cuc et al., 2006; Stone et al., 2010).

The goal of the present study was to address to what degree collaborative facilitation and social contagion might also serve a directive function, and might help to inform and guide future individual behavior (Bluck, 2003; Bluck et al., 2005; Pillemer, 2003, 2009). In particular, in situations where socially transmitted information is useful for subsequent tasks, effects of collaborative remembering and the integration of information from social sources could serve to improve individual behavior in these tasks, making an individual’s behavior more adaptive. Potentially, to the degree that behavior is influenced by socially transmitted information, and similar to how collaborative remembering has been shown to contribute to the development of collectively held memories, collaborative remembering might even synchronize subsequent behavior such that individuals later act more similarly.

### Memory and adaptive decision making

To address a potential directive function of effects of collaborative remembering, the present study made use of a modified version of a prisoner’s dilemma. This specific task was chosen because prior work has demonstrated a relationship between individual memory and adaptive decision making in this task (Schaper et al., 2019). Generally, in this task, participants are asked to decide whether they want to cooperate with players whose faces are presented on a screen. Following a typical pay-off matrix (for details, see Schaper et al., 2019, p. 9), participants are informed that different consequences arise, depending on their own as well as the player’s decision. If participant and the player on the screen cooperate, each receives a net bonus of 10 cents. If only one of them cooperates and the other one cheats, the person who cooperated takes a net loss of −10 cents, whereas the person who cheated takes a net gain of 20 cents. If nobody cooperates, nothing happens (neither gains nor losses).

Schaper et al. (2019) further modified this task to incorporate three phases. In an initial exposure phase, participants encounter the faces of several players and learn whether they are cooperators or cheaters. To achieve this, participants are not free to make their own decisions in this phase, but are forced to always cooperate with the players, whereas the players can reciprocate the participant’s cooperation (thus acting as cooperators) or not reciprocate it (thus acting as cheaters). The second phase is an interpolated memory test. Here, the faces of all players from the exposure phase are presented again, randomly intermixed with new faces. Participants are now asked to judge for each face whether it is an old face from the exposure phase, and if so, whether the face belonged to a cooperator or cheater. The third phase is a final decision game, in which participants can try to maximize their own gains. In this phase, participants again encounter faces of old and new players, but are now given free choice to decide whether they want to cooperate with each single player or not.

Applying this task in individuals, Schaper et al. (2019) found that participants were most likely to cooperate with cooperators from the initial exposure phase during the final decision game. Cooperation rates with cheaters from the initial exposure phase were much lower and in fact at the same level as cooperation rates with completely new players. This pattern was interpreted as reflecting adaptive decision behavior, inasmuch as higher cooperation rates with cooperators increase gains, and lower cooperation rates with cheaters decrease losses. Importantly, source memory performance on the interpolated memory test was positively correlated with cooperation rates with cooperators, as well as a negatively correlated with cooperation rates with cheaters, suggesting a link between memory and adaptive decision making. In particular, these findings are consistent with the notion that memory for previous interactions supports direct reciprocal cooperation (Bell et al., 2010; Buchner et al., 2009). Further studies in the literature used different tasks, but also support a link between episodic memory and adaptive decisions. For example, Murty et al. (2016) showed that associative memories for reward values inform adaptive decision making in a lottery game as well as a in a dictator game (see also FeldmanHall et al., 2021). Rasmussen and Gutchess (2019) found a connection between memory and investment decisions in younger and older adults. Kroneisen et al. (2021) also reported a connection between memory and subsequent investment decisions in a two-player public goods game.

In sum, this work suggests that memories can direct later decision making. An open question is whether this is only the case for memories based on first-hand experiences, or whether memories based on socially transmitted information can serve the same function. To the degree that socially shared information is relevant for subsequent decisions, it would be adaptive for individuals to use the information to optimize their own decision making. Individuals may not always possess all relevant information, and the integration of so far unknown, but relevant information from social sources could help promote adaptive behavior in the present and future. This would be largely consistent with a framework focused on the adaptive nature of memory (e.g., Klein, 2013; Nairne, 2010).

### The present study

The present study borrowed the task that was introduced by Schaper et al. (2019), with slight alterations in order to be able to examine the potential role of collaborative remembering for subsequent decision making. Participants completed the task in groups of three. The initial exposure phase and the final decision game were always completed individually by all participants, but for the interpolated memory test, it was manipulated whether participants completed this test individually as well or had the opportunity to freely interact with the other group members while working on the same test as a group. The main hypothesis was that the opportunity for collaboration during the interpolated memory test should enable collaborative facilitation for initially studied as well as social contagion with initially unstudied information, thus potentially improving subsequent individual decision making on the final decision game. To this end, during the initial exposure phase, only some of the faces that participants encountered on the screen were the same across the three group members; other faces were only encountered by single group members.

If effects of collaborative remembering can serve a directive function and guide subsequent behavior, collaborative facilitation for initially studied information should increase adaptive decision making for faces of cooperators and cheaters that were directly encountered during the initial exposure phase. In addition, social contagion with initially unstudied information should also increase adaptive decision making for faces of cooperators and cheaters that were initially only encountered by other group members, but not by oneself. Taken together, if effects of collaborative remembering increase adaptive decision making, then participants from collaborative groups should be more likely to cooperate with cooperators than with cheaters or completely new players, irrespective of whether these players were directly encountered or encountered by other group members only. In three experiments, these hypotheses were put to the test.

## Experiment 1

### Method

#### Participants

Ninety subjects participated in groups of three, with 15 triads (*n* = 45) in each of 2 experimental conditions (collaborative vs. nominal groups). When signing up, subjects knew that they would complete the experiment together with two other participants. Mean age was 24.5 years ( SD=3.7 ); 59 subjects were female, 31 subjects were male. None of the participants knew each other before the experiment.

The sample size was chosen based on prior work that had demonstrated effects of collaboration on subsequent individual memory (e.g., Abel & Bäuml, 2020; Blumen & Stern, 2011; Blumen et al., 2014; Weldon & Bellinger, 1997). A sensitivity analysis for ANOVAs with repeated measures (for 2 groups and 3 measurements) was conducted in G*Power (Faul et al., 2007). With power set to .80, alpha to .05, and correlations among repeated measures to .50, the analysis suggested that this sample size enabled the detection of medium-sized between-subjects effects ( f=0.24 ) and smaller-sized within-subject as well as interaction effects ( f=0.14 ).

#### Material

Sixty faces were selected from the Face Research Lab London Set (DeBruine & Jones, 2017) and were divided into 5 sets of 12 faces each, with half of the faces in each set being male/female. Three of the five sets were used as to-be-studied material during the initial exposure phase of the experiment and assigned to be studied by all three, two, or one of the group members (i.e., they served as fully shared, partly shared, or unshared materials; see Abel & Bäuml, 2020, 2023, as well as the more detailed description in the next section). The remaining two sets were used as distractor materials for the interpolated memory test or the final decision game, respectively. Materials were counterbalanced across participants, that is, each set served equally often as fully shared, partly shared, or unshared study materials, or as distractor materials for the memory test or decision game.

#### Design

The main experiment had a three-factorial mixed design. The first factor of group type (nominal vs. collaborative) was manipulated between-subjects. All subjects participated in groups of three, but in the nominal group condition, subjects completed an interpolated memory test alone, without interacting with the other group members. In the collaborative group condition, subjects engaged in the same interpolated test, but completed it collaboratively, while freely interacting with the other group members.

The second factor of player type (cooperator, cheater, new) was manipulated within-subject. During an initial exposure phase, some of the faces that participants saw acted as cooperators (i.e., they reciprocated the participants’ cooperation, resulting in a net gain for the participants), whereas others acted as cheaters (i.e., they did not reciprocate, resulting in a net loss for the participants). In later phases of the experiment, faces of cooperators and cheaters were shown intermixed with faces of completely new players that had not been encountered by anyone in the group.

Finally, the third factor of information distribution was manipulated within-subject. During the initial exposure phase, only some faces of cooperators and cheaters were encountered by all three group members (fully shared), whereas other cooperators and cheaters were encountered by two group members (partly shared) or one group member only (unshared). This manipulation allows one to discriminate between players that were directly encountered—and players that were encountered by other group members only, but not by oneself. The main interest of this study was to examine if interpolated collaborative remembering serves social information transmission and improves adaptive decision making. Adaptive decision making was analyzed at two levels. First, with regard to cooperators and cheaters that were directly encountered before, and second, with regard to cooperators and cheaters that were only encountered by other group members. Additionally, it would be possible to also differentiate between initial information distribution (i.e., whether faces were initially seen by one, two, or all three group members). For brevity, this additional differentiation is neglected in this study, but interested readers are invited to consult the data files provided on the Open Science Framework for further details (https://osf.io/mcj7k/).

#### Procedure

Due to the COVID-19 pandemic and the restrictions in place at the time, the experiments reported in this study could not be conducted in the lab and were run online via the video conference software Zoom instead. Importantly, prior work on the collaborative recognition task has shown similar findings, irrespective of whether collaborative remembering occurred in the lab or online via Zoom (Abel & Bäuml, 2023; for further work suggesting similar effects in synchronous online settings, see Conte et al., 2023; Rossi-Arnaud et al., 2023). Subjects participated from their homes and were invited to a joint Zoom meeting via e-mail. Three experimenters were necessary to simultaneously conduct the study with three participants, partly in individual break-out sessions (see below for further details). Before the start of the experiment, participants received basic information about the study and their rights as participants; all provided verbal consent to participate in the study. They were asked to keep their cameras and microphones activated to enable communication and social exchange. For privacy reasons, no video or audio recordings were made, and this aspect was also emphasized to participants.

##### Initial exposure phase

The initial exposure phase was completed individually by all participants. Each subject was accompanied to a separate break-out session by an experimenter, who shared their screen for stimulus presentation. During the initial exposure phase, each participant encountered 24 players, half of which were cooperators, the other half cheaters. The faces of these players were presented in random order, centrally on the screen, in front of a gray background. The general task set-up was a modified version of a prisoner’s dilemma and closely based upon prior work that established a relationship between individual memory and adaptive decision making (Schaper et al., 2019). Participants were informed about the basic task structure and payoff matrix (for details, see Schaper et al., 2019), with reciprocal cooperation decisions resulting in a net gain of 10 cents for each player and one-sided cooperation resulting in a net loss of −10 cents for the cheated player (but a net gain of 20 cents for the cheating player). Participants were aware that they would only be able to cooperate with all players during the initial exposure phase, but that they would have the opportunity to make their own decisions (i.e., to cooperate or not) in a later phase of the experiment.

On each trial of the initial exposure phase, a player’s face was first presented for 3 s centrally on the screen, before the text “Your decision: . . .” appeared below the face for 1 s in a white font, followed by “Your decision: You cooperate” for 1 s. If the player was a cooperator, they reciprocated the participant’s cooperation; if the player was a cheater, they did not reciprocate. The text “Your partner’s decision: . . .” was presented in blue font below the face for 1 s, followed by “Your partner’s decision: He/She cooperates” or “Your partner’s decision: He/She does NOT cooperate” for 2 s. Next, participants were informed about the consequences of the partner’s decision. If they had encountered a cooperator, the text “Your gain: + 10 cents” was shown in white font below the face for 2 s; if they had encountered a cheater, the text instead said “Your loss: −10 cents.” Finally, an updated account balance was shown centrally on the screen for 2 s, before the next trial started.

Each participant in a triad encountered 24 faces in the exposure phase, but only 12 of these faces were in fact seen by all 3 group members; 8 of the faces were seen by the participant and 1 other group member, and 4 were encountered by the respective participant only. Half of the faces would typically be identified as male, and the other half as female; moreover, half of the players acted as cooperators, and the others as cheaters. Across all 3 group members, a total of 36 faces were encountered (12 were encoded by all 3 participants, 12 were encoded by 2 participants, and 12 were encoded by 1 participant; for further details on this manipulation, see Abel & Bäuml, 2020, 2023).

##### Distractor phase

Afterward, participants returned to the main Zoom meeting and were distracted for 15 min. First, they provided demographic information about themselves, which took roughly 2 min. For the remaining time, they were asked to complete standard progressive matrices (Raven, 2000).

##### Interpolated collaborative versus individual memory test

On the interpolated memory test, all 36 faces that had been encountered by at least 1 group member were presented again, back to back, centrally on the screen, and randomly intermixed with 12 new distractor faces. For each face, subjects were asked to decide if they had encountered the respective player during the initial exposure phase or not. Only if they responded that they had encountered the player were they next asked to also make a source memory judgment with regard to a player’s past behavior and to indicate if the player was a cooperator or a cheater.

In collaborative groups, subjects completed the interpolated test together, in the main Zoom meeting. They were asked to decide as a group if they had encountered the players initially (and if so, whether they were cheaters or cooperators). Subjects were given no specific instructions on how to determine group responses and were allowed to freely interact with each other. The test was self-paced, and faces plus response options stayed on the screen until the group’s response was entered by the experimenter via the computer keyboard.

Subjects in the nominal group condition completed the same test, but did so alone and in individual break-out rooms, without any social interaction with the other group members. After completing the test, subjects in all conditions worked on a series of problem-solving tasks for 5 min in individual break-out rooms.

##### Final decision game

The final decision game was also completed individually and in break-out rooms by subjects in all conditions. In this game, they were again presented with the 36 faces that had been encountered by at least 1 group member in the initial exposure phase, and the faces were again presented randomly intermixed with 12 completely new distractor faces, not previously encountered by anyone in the group. On each trial, participants could choose freely whether they wanted to cooperate with the respective player on the screen or not. Each face stayed on the screen until the participant’s response was recorded by the experimenter. Following Schaper et al. (2019), during this phase of the experiment, participants did not receive any feedback regarding the players’ behavior.

When the final decision game was completed, participants moved on to a self-paced source memory test. They again saw the 48 faces from the decision game in a random order and were asked to indicate for each face whether they remembered encountering it during the initial exposure phase only (response option 1), the initial exposure phase and the interpolated memory test (response option 2), the interpolated memory test only (response option 3), or in none of the previous phases of the experiment (response option 4).^1^ Upon completion, subjects returned to the main Zoom meeting for goodbyes, where they were also debriefed and thanked for their participation. Participants received partial course credit or an online gift voucher in return for their participation.

### Results

#### Individual cooperation behavior during the final decision game

**Figure 1.**
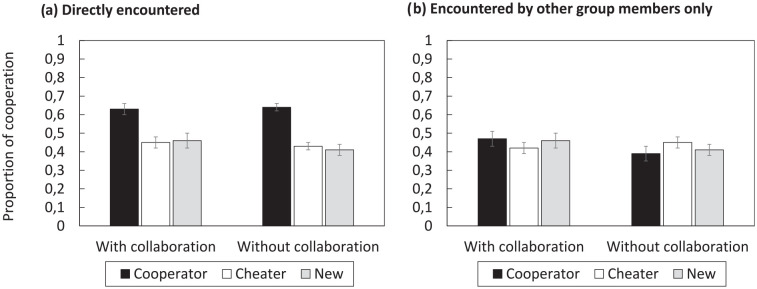
Mean proportion of decisions to cooperate in Experiment 1. (a) Shows cooperation rates with players directly encountered during the initial study phase; (b) shows cooperation rates with players that were encountered by other group members only. Error bars represent ±1 standard error of the mean.

The first analysis focused on cooperation decisions for players that were directly encountered in the initial study phase (see Figure 1a for mean proportions of cooperation decisions). A 2 × 3 ANOVA with the factors of GROUP CONDITION (nominal, collaborative) and PLAYER TYPE (cooperator, cheater, new) showed a significant main effect of PLAYER TYPE, F(1.77,155.85)=33.45 , MSE=0.04 , p<.001 , $\eta_{p}^{2}=.28$ , but no significant main effect of GROUP CONDITION, F(1,88)=0.34 , MSE=0.05 , p=.561 , $\eta_{p}^{2}=.004$ , and also no significant interaction between the two factors, F(1.77,155.85)=0.63 , MSE=0.04 , p=.514 , $\eta_{p}^{2}=.007$ . The pattern of decisions to cooperate with different player types was highly similar, irrespective of whether participants were given an opportunity for interpolated collaboration or not. In both nominal and collaborative groups, participants cooperated significantly more with players who had previously cooperated with them than with new players ( t(89)=6.43 , p<.001 , d=0.68 ) or with players who had previously cheated them ( t(89)=8.77 , p<.001 , d=0.93 ). Cooperation rates did not differ significantly for new players and cheaters ( t(89)=0.09 , p=.925 , d=0.01 ).

The second analysis focused on cooperation decisions for players which were initially encountered by other group members only, not by oneself (see Figure 1b for mean cooperation rates). A 2 × 3 ANOVA parallel to the one reported above showed no significant main effect of PLAYER TYPE, F(2,176)=0.03 , MSE=0.03 , p=.970 , $\eta_{p}^{2}<.001$ , no significant main effect of GROUP CONDITION, F(1,88)=0.74 , MSE=0.10 , p=.392 , $\eta_{p}^{2}=.008$ , and also no significant interaction between the two factors, F(2,176)=2.59 , MSE=0.03 , p=.078, $\eta_{p}^{2}=.03$ . Decisions to cooperate with players who had previously cooperated with or cheated other group members did not differ significantly from decisions to cooperate with completely new players. Critically, this was not just the case in the nominal control condition where this pattern would be expected ( F(1.64,71.95)=1.53 , MSE=0.04 , p=.225 , $\eta_{p}^{2}=.03$ ), but also in the condition with prior collaboration and an actual opportunity for information exchange between group members ( F(2,88)=1.12 , MSE=0.03 , p=.331 , $\eta_{p}^{2}=.03$ ).

To address nonsignificant findings, additional Bayesian repeated-measures ANOVAs were conducted in JASP (Version 0.17.3; JASP Team, 2023; see also van den Bergh et al., 2020). The results of these analyses were consistent with those reported above. For decisions toward directly encountered players, there was strong evidence for an effect of player type ( $BF_{\mathrm{incl}}=9.60\;\times \;10^{10}$ ), but moderate-to-strong evidence against an effect of group condition ( $BF_{\mathrm{incl}}=0.14$ ) and against an interaction effect ( $BF_{\mathrm{incl}}=0.08$ ). For decisions toward players that were encountered by other group members only, there was moderate-to-strong evidence against effects of player type ( $BF_{\mathrm{incl}}=0.03$ ), and group condition ( $BF_{\mathrm{incl}}=0.21$ ), as well as the interaction effect ( $BF_{\mathrm{incl}}=0.02$ ).

##### Performance on the interpolated collaborative versus individual test

One reason for why collaborative remembering did not promote adaptive decision making might be that information transmission during the interpolated collaborative test was not successful. To address this possibility, performance on the interpolated test was analyzed. In particular, collaborative triads should show much higher performance on this test, because all 36 players from the initial exposure phase were encountered by at least one of the group members. Without a chance for social information sharing, individuals should only be able to correctly recognize the players that they themselves encountered during the exposure phase (i.e., a maximum of 24 out of the 36 players). In other words, comparing group performance in the collaboration condition to individual performance in the control condition serves as a rough manipulation check to evaluate the degree of social information sharing during collaboration. To equate numbers of observations, the group scores of the 15 collaborative triads were compared to the individual scores of 15 randomly drawn participants from the control condition. Note that, due to this focus on analyses at the group level, sample size was lower than for comparisons at the individual level. A sensitivity analysis ( 1−β=.80 , α=.05 , corr=.50 ) suggested that the sample size enabled the detection of large-sized between-subjects effects ( f=0.46 ), medium-sized within-subject effects ( f=0.27 ), and smaller-sized interaction effects ( f=0.15 ).

**Figure 2.**
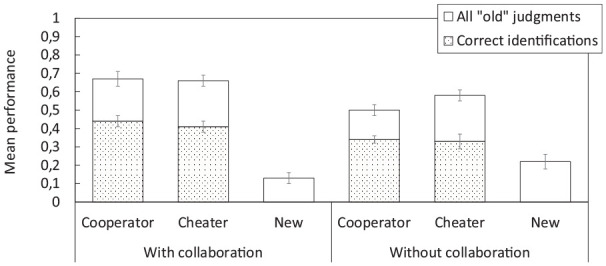
Mean performance on the interpolated memory test (with vs. without collaboration) in Experiment 1. The stacked bars refer to all “old” judgments during the old/new recognition part of the test; the dotted bars refer to correct player identifications only (out of all cooperators or cheaters, respectively). Error bars represent ±1 standard error of the mean.

Figure 2 shows mean performance on the interpolated test. Collaborative groups judged significantly more players from the initial study phase as old than participants who took the test alone ( M=0.66 vs. M=0.54 ; F(1,28)=8.87 , MSE=0.03 , p=.006 , $\eta_{p}^{2}=.24$ ). Hit rates for cooperators and cheaters did not differ significantly ( F(1,28)=1.69 , MSE=0.01 , p=.204 , $\eta_{p}^{2}=.06$ ), and the interaction between player type and group condition was also not significant ( F(1,28)=3.09 , MSE=0.01, p=.090 , $\eta_{p}^{2}=.10$ ). False alarms for new players were lower in collaborative groups than in individuals ( M=0.13 vs. M=0.22 ), but the difference was not statistically significant ( t(28)=1.85 , p=.075 , d=0.68 ).

Although collaborative groups successfully identified more players from the study phase as old, they would also need to correctly identify them as cheaters or cooperators in order for collaboration to potentially benefit later individual decision making. Looking at correct player attributions only, collaborative groups still identified more players correctly as cooperators or cheaters than individuals. Yet, this difference between group conditions seemed less pronounced, and correct attribution rates were not particularly high ( M=0.42 vs. M=0.34 ; F(1,28)=4.51 , MSE=0.02 , p=.043 , $\eta_{p}^{2}=.14$ ). There was again no difference for correct identifications of cooperators and cheaters ( F(1,28)=0.56 , MSE=0.01 , p=.459 , $\eta_{p}^{2}=.02$ ), and the interaction between player type and group condition was also not significant ( F(1,28)=0.12 , MSE=0.01 , p=.735 , $\eta_{p}^{2}=.004$ ).^2^

### Discussion

The results in the individual control condition without social exchange replicate the findings by Schaper et al. (2019). Having encountered cheaters and cooperators in an initial exposure phase, individuals were more likely to later cooperate with cooperators than with cheaters or new players. Importantly, the same pattern was found in the collaborative condition, with interpolated social exchange and collaborative remembering. Contrary to expectations, collaborative remembering did, however, not result in enhanced adaptive decision making—neither regarding players that were directly encountered in the initial exposure phase, nor regarding players that were initially encountered by the other group members. Collaborative remembering did, therefore, not seem to serve a directive function and guide later individual decision making.

An analysis of the interpolated collaborative versus individual test showed that collaborative groups were more successful at identifying players from the study phase as old, and were also slightly more successful at correctly identifying them as cooperators or cheaters. Overall success rates on this test were, however, not particularly high in either group condition, which might suggest that participants in the collaboration condition were not able to socially share enough critical information about former cooperators and cheaters in this phase.

## Experiment 2

Experiment 2 was conducted to address if collaborative remembering might enhance adaptive decision making if individuals were better equipped to actually transmit critical information. In particular, in Experiment 1, the correct identification rates of cooperators and cheaters during the interpolated collaborative test, though not at floor levels, were not particularly high (0.42 vs. 0.34 with and without collaboration, respectively). Less than 50% of all players encountered by anyone in the group were successfully identified as cooperators or cheaters. Correct identifications are, however, essential for adaptive decisions in this task, and at least potentially, the observed 8% difference between group conditions may have been too small to have a meaningful effect on later individual decision making.

In Experiment 2, to improve performance on the interpolated collaborative test, two changes were made to the experimental procedure. During the initial exposure phase, participants now saw each face (and the corresponding behavior) twice, which should support participants in associating specific faces with specific behaviors (cooperating vs. cheating). Moreover, the retention interval between initial exposure phase and interpolated test was reduced from 15 to 5 min. Both changes should improve performance on the interpolated test and benefit the transmission of critical information in collaborative groups.

### Method

#### Participants

90 subjects participated in groups of three (with 15 groups per condition; see Experiment 1 for a sensitivity analysis). Mean age was 23.2 years ( SD=2.8 ); 38 subjects were male and 52 subjects were female. None of the participants knew each other before the experiment.

#### Material and design

Materials and design were the same as in Experiment 1.

#### Procedure

The procedure of the study was identical to that of Experiment 1, except for two changes. The first change concerned the initial exposure phase, where all faces were no longer presented once, but twice. After a first study cycle and presentation of all faces, a second study cycle began and all faces were presented again and in a new random order. Faces were always associated with the same behavior (cooperating vs. cheating) across the two study cycles. The second change concerned the distractor phase between initial exposure and the interpolated memory test. Instead of 15 min, participants were now only distracted for 5 min. After providing basic demographic information about themselves, participants completed Standard Progressive Matrices (Raven, 2000) for the remaining time. All other procedural details were identical to Experiment 1.

### Results

#### Individual cooperation behavior during the final decision game

**Figure 3.**
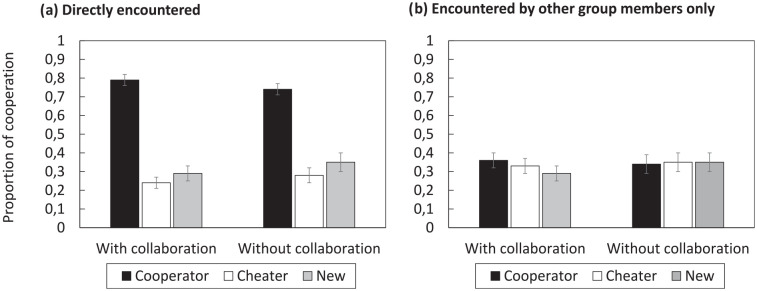
Mean proportion of decisions to cooperate in Experiment 2. (a) Shows cooperation rates with players directly encountered during the initial study phase; (b) shows cooperation rates with players that were encountered by other group members only. Error bars represent ±1 standard error of the mean.

The first analysis focused on cooperation decisions for players directly encountered in the initial study phase (see Figure 3a). The pattern of results resembled Experiment 1, but differences between player types were even more pronounced in Experiment 2. A 2 × 3 ANOVA with the factors of GROUP CONDITION (nominal, collaborative) and PLAYER TYPE (cooperator, cheater, new) showed a significant main effect of PLAYER TYPE, F(2,176)=130.29 , MSE=0.05 , p<.001 , $\eta_{p}^{2}=.60$ , but no significant main effect of GROUP CONDITION, F(1,88)=0.24 , MSE=0.07 , p=.628 , $\eta_{p}^{2}=.003$ , and also no significant interaction between the two factors, F(2,176)=1.59 , MSE=0.05 , p=.208 , $\eta_{p}^{2}=.02$ . In both nominal and collaborative groups, participants were more likely to cooperate with cooperators than with new players ( t(89)=11.70 , p<.001 , d=1.23 ) or with cheaters ( t(89)=15.84 , p<.001 , d=1.67 ). Cooperation rates did again not differ significantly for new players and cheaters ( t(89)=1.87 , p=.065 , d=0.20 ).

The second analysis focused on cooperation decisions for players initially encountered by other group members only (see Figure 3b). The pattern of results replicated Experiment 1. A 2 × 3 ANOVA showed no significant main effect of PLAYER TYPE, F(2,176)=0.71 , MSE=0.02 , p=.494 , $\eta_{p}^{2}=.01$ , no significant main effect of GROUP CONDITION, F(1,88)=0.13 , MSE=0.24 , p=.718 , $\eta_{p}^{2}=.001$ , and also no significant interaction between the two factors, F(2,176)=1.51 , MSE=0.02 , p=.224 , $\eta_{p}^{2}=.02$ . Decisions to cooperate with players who had previously cooperated with or cheated other group members did again not differ significantly from decisions to cooperate with completely new players—neither in the nominal control condition ( F(2,88)=0.10 , MSE=0.02 , p=.905 , $\eta_{p}^{2}=.002$ ), nor in the collaborative condition with an opportunity for social information exchange ( F(1.72,75.54)=2.00 , MSE=0.03 , p=.149 , $\eta_{p}^{2}=.04$ ).

Additional Bayesian ANOVAs were conducted to address nonsignificant findings; the results were consistent with those reported above. For decisions toward directly encountered players, there was very strong evidence for an effect of player type ( $BF_{\mathrm{incl}}$ = ∞ ), but moderate evidence against an effect of group condition ( $BF_{\mathrm{incl}}=0.16$ ) as well as the interaction effect ( $BF_{\mathrm{incl}}=0.18$ ). For decisions toward players that were encountered by other group members only, there was strong evidence against an effect of player type ( $BF_{\mathrm{incl}}=0.05$ ) and the interaction effect ( $BF_{\mathrm{incl}}=0.02$ ), as well as moderate evidence against an effect of group condition ( $BF_{\mathrm{incl}}=0.25$ ).

##### Performance on the interpolated collaborative versus individual test

**Figure 4.**
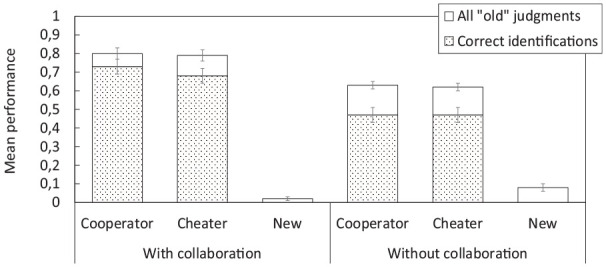
Mean performance on the interpolated memory test (with vs. without collaboration) in Experiment 2. The stacked bars refer to all “old” judgments during the old/new recognition part of the test; the dotted bars refer to correct player identifications only (out of all cooperators or cheaters, respectively). Error bars represent ±1 standard error of the mean.

The procedural changes made in Experiment 2 had the goal to increase interpolated test performance, and Figure 4 suggests that the changes were effective. False alarms for new players were significantly lower in collaborative groups than in individuals ( M=0.02 vs. M=0.08 ; t(28)=2.91 , p=.007 , d=1.06 ). Collaborative groups, however, judged significantly more players from the initial study phase as old than participants who took the test alone ( M=0.80 vs. M=0.62 ; F(1,28)=23.81 , MSE=0.02 , p<.001, $\eta_{p}^{2}=.46$ ). Hit rates for cooperators and cheaters did not differ significantly ( F(1,28)=0.97 , MSE=0.003 , p=.334, $\eta_{p}^{2}=.03$ ), and the interaction between player type and group condition was also not significant ( F(1,28)=0.02 , MSE=0.003 , p=.889 , $\eta_{p}^{2}=.001$ ).

Collaborative groups were also more successful than individuals in correctly identifying old players as cooperators or cheaters, respectively ( M=0.71 vs. M=0.47 ; F(1,28)=21.49 , MSE=0.04 , p<.001 , $\eta_{p}^{2}=.43$ ). Note that rates of successful attributions were higher in Experiment 2 than in Experiment 1, especially in collaborating triads. There was again no significant difference for the identification of cooperators and cheaters ( F(1,28)=2.03 , MSE=0.01 , p=.165 , $\eta_{p}^{2}=.07$ ), and the interaction between player type and group condition was also not significant ( F(1,28)=1.09 , MSE=0.01 , p=.305 , $\eta_{p}^{2}=.04$ ).

### Discussion

The procedural changes implemented in Experiment 2 were effective and enhanced performance on the interpolated collaborative test. In particular, successful identification of cooperators and cheaters was now at 71% correct in collaborative groups, and around 24% higher relative to the control condition. Yet, despite enhanced performance on the interpolated collaborative test, the main results of Experiment 2 replicated those of Experiment 1. Participants in both group conditions showed adaptive decision behavior toward players that they had directly encountered in the initial exposure phase. Interpolated collaborative remembering did, however, not further improve individual decision making—neither for directly encountered players nor for players encountered by other group members only.

## Experiment 3

Why did participants in Experiment 2 not use socially shared information to optimize their own decision behavior? One reason could still be located in the interpolated test. In both Experiments 1 and 2, the interpolated test was carried out in two steps. For each face, participants first provided an old/new judgment, which was followed by identifying an old player as cheater or cooperator. This separation might have tempted participants to ignore the second judgment if they themselves did not remember having encountered the respective player. Since this is a critical piece of information for later adaptive decisions, its neglect could potentially account for why participants were later not able to use the information. Therefore, in Experiment 3, the format of the interpolated memory test was changed, and old/new judgment and player identification were merged into one step.

Another issue might however also be that participants were simply not motivated to try and benefit from collaboration. For example, they may have become skeptical when other group members remembered players that they did not remember. To address this possibility, Experiment 3 not only included a collaboration condition with regular instructions (as in Experiments 1 and 2), but also an additional collaboration condition with adapted, inclusive instructions. Following prior work on collaborative remembering that had varied instructions during collaboration (Jalbert et al., 2021; see also Hyman et al., 2014), participants with inclusive instructions were asked to include all faces as old that were initially encountered by anyone in the group. With inclusive instructions, participants were also debriefed about the fact that group members had initially encountered partly different faces, and that the collaboration phase might enable them to learn more about other players. No such debriefing was present in the regular collaboration condition, the idea being that participants with inclusive instructions should be more motivated to try to benefit from socially shared information.

### Method

#### Participants

A total of 180 subjects participated in the experiment in groups of three. Mean age was 25.3 years ( SD=4.1 ); 61 subjects self-identified as male, 118 as female, and 1 as diverse. None of the participants in each triad were friends or well acquainted before the experiment.

The number of triads per condition (*n* = 20; 60 individuals) was enhanced relative to the previous experiments, because the factor of group condition now included three factor levels. The overall sample size corresponded to the maximum number of participants that could be tested at the time. A sensitivity analysis for ANOVAs with repeated measures (for 3 groups and 3 measurements) was conducted in G*Power (Faul et al., 2007). With power set to .80, alpha to .05, and correlations among repeated measures to .50, the analysis suggested that this sample size enabled detection of medium-sized between-subjects effects ( f=0.19 ) and smaller-sized within-subject effects (*f* = 0.10) as well as smaller-sized interaction effects ( f=0.11 ).

#### Material

Materials were identical to Experiments 1 and 2.

#### Design

The design of Experiment 3 was the same as for the previous experiments, but the factor group condition now had three factor levels (nominal, collaborative without specific instructions, and collaborative with inclusive instructions).

#### Procedure

The procedure of Experiment 3 was the same as for Experiment 2, except that the format of the interpolated collaborative test was adapted and an additional collaboration condition with inclusive instructions was included. On the interpolated memory test, participants were now asked to indicate for each face whether it was an old player from the initial exposure phase that had acted as a collaborator, an old player that had acted as a cheater, or a new player that they had not encountered before (thus merging the previously separated old/new judgment and player identification into a single step).

In the nominal and the collaborative group condition with regular instructions, no further changes were made. With inclusive instructions, participants were, however, debriefed about the unequal distribution of faces during the initial exposure phase before they started the interpolated test, and it was suggested that the interpolated test might provide an opportunity to learn about other players in the decision game, which they had not encountered themselves yet. Moreover, participants were asked to include all old players on this test, irrespective of how many group members had encountered them during the initial study phase.

### Results

#### Individual cooperation behavior during the final decision game

**Figure 5.**
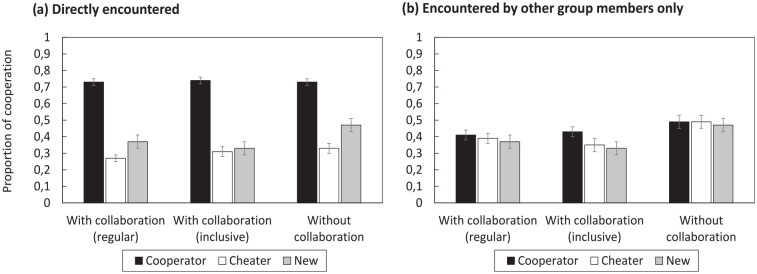
Mean proportion of decisions to cooperate in Experiment 3. (a) Shows cooperation rates with players directly encountered during the initial study phase; (b) shows cooperation rates with players that were encountered by other group members only. Error bars represent ±1 standard error of the mean.

Figure 5a shows cooperation decisions for players directly encountered in the initial study phase. A 3 × 3 ANOVA with the factors of GROUP CONDITION (nominal, collaborative without specific instructions, collaborative with inclusive instructions) and PLAYER TYPE (cooperator, cheater, new) showed a significant main effect of PLAYER TYPE, F(1.93,342.19)=193.99 , MSE=0.05 , p<.001 , $\eta_{p}^{2}=.52$ , as well as a significant main effect of GROUP CONDITION, F(2,177)=3.06 , MSE=0.05 , p=.049 , $\eta_{p}^{2}=.03$ , whereas the interaction between the two factors did not reach significance, F(3.38,342.19)=2.11 , MSE=0.05 , p=.082 , $\eta_{p}^{2}=.02$ . Participants were more likely to cooperate with cooperators than with new players ( t(179)=13.59 , p<.001 , d=1.01 ) or with cheaters ( t(179)=20.41 , p<.001 , d=1.52 ); they were also less likely to cooperate with cheaters than with new players ( t(179)=3.81 , p<.001 , d=0.28 ). A comparison of the two conditions with collaboration showed no significant main or interaction effects involving the factor GROUP CONDITION, Fs≤0.99 , ps≥.365 , $\eta_{p}^{2}\leq .01$ . Yet, there was a significant main effect for GROUP CONDITION when contrasting the collaborative condition without specific instructions to the nominal condition, F(1,118)=7.25 , MSE=0.04 , p=.008 , $\eta_{p}^{2}=.06$ , and a significant interaction effect between GROUP CONDITION and PLAYER TYPE when contrasting the collaborative condition with inclusive instructions to the nominal condition, F(2,236)=4.10 , MSE=0.05 , p=.018 , $\eta_{p}^{2}=.03$ . Mean cooperation with new players was higher in the control condition without collaboration relative to the collaboration condition with inclusive instructions, *t*(118) = 2.64, p=.010 , d=0.48 ; there were no further significant differences, ts(118)=0.36 , *p*s = .723, ds=0.07 .

Figure 5b shows cooperation decisions for players initially encountered by other group members only. A 3 × 3 ANOVA showed a significant main effect of PLAYER TYPE, F(2,354)=4.57 , MSE=0.03 , p=.011 , p=.003 , a significant main effect of GROUP CONDITION, F(2,177)=4.00 , MSE=0.17 , p=.020 , $\eta_{p}^{2}=.04$ , but no significant interaction between the two factors, F(4,354)=0.95 , MSE=0.03 , p=.433 , $\eta_{p}^{2}=.01$ . Overall, participants were slightly more likely to cooperate with cooperators that had initially been encountered by other group members than with completely new players ( t(179)=3.06 , $\eta_{p}^{2}=.48$ , d=0.23 ); cooperation rates did not differ for new players and cheaters encountered by other group members, or between cooperators and cheaters encountered by other group members ( ts(179)≤1.81 , ps≥.072 , ds≤0.14 ). Again, the two collaboration conditions did not differ significantly, Fs≤1.01 , ps≥.366 , $\eta_{p}^{2}\leq .01$ . Yet, there were significant main effects for GROUP CONDITION when contrasting each of the two collaboration conditions with the nominal group condition, *F*(1,118) ≥ 482, ps≤.030 , $\eta_{p}^{2}\geq .04$ . Cooperation rates were higher in the control condition relative to both collaboration conditions.

Again, additional Bayesian analyses were conducted. For decisions toward directly encountered players, there was very strong evidence for an effect of player type ( $BF_{\mathrm{incl}}=3.34\times 10^{14}$ ), but moderate evidence against the interaction effect ( $BF_{\mathrm{incl}}=0.30$ ) as well as against an effect of group condition ( $BF_{\mathrm{incl}}=0.27$ ); the last result contrasts with the ANOVA reported above. For decisions toward players that were encountered by other group members only, there was strong evidence against an interaction effect ( $BF_{\mathrm{incl}}=0.07$ ). The evidence regarding an effect of player type was inconclusive ( $BF_{\mathrm{incl}}=0.99$ ), and the evidence for an effect of group condition was anecdotal ( $BF_{\mathrm{incl}}=1.67$ ); these two findings contrast with the ANOVA results reported above.

##### Performance on the interpolated collaborative versus individual test

**Figure 6.**
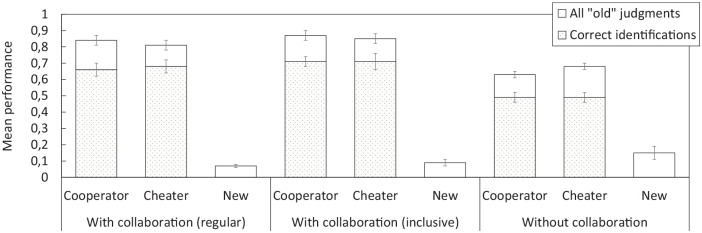
Mean performance on the interpolated memory test (with vs. without collaboration) in Experiment 3. The stacked bars refer to all “old” judgments during the old/new recognition part of the test; the dotted bars refer to correct player identifications only (out of all cooperators or cheaters, respectively). Error bars represent ±1 standard error of the mean.

False alarms for new players did not differ significantly across the three group conditions, even though they were numerically higher in the nominal group condition without collaboration ( M=0.07 vs. M=0.09 vs. M=0.15 ; F(1,57)=2.19 , MSE=0.02, p=.121, $\eta_{p}^{2}=.07$ ; see Figure 6). A 2 × 3 ANOVA on hit rates did not show a significant main effect of player type ( F(1,57)=0.02, MSE=0.01 , p=.885 , $\eta_{p}^{2}<.001),$ but there was a significant main effect of group condition, ( F(2,57)=26.00, MSE=0.02 , p<.001, $\eta_{p}^{2}=.48$ ), which was accompanied by a significant interaction between the two factors ( F(2,57)=4.31 , MSE=0.01 , p=.018 , $\eta_{p}^{2}=.13).$ Hits for cheaters and cooperators did not differ in the two collaborative group conditions ( Fs(1,19)≤3.06 , ps≥.097 , $\eta_{p}^{2}\leq .14$ ), but in the control condition without collaboration, hit rates were significantly higher for cheaters than for cooperators ( F(1,19)=5.52 , MSE=0.01 , p=.030, $\eta_{p}^{2}=.23$ ). Most importantly, hit rates did not differ between the two collaborative conditions ( F(1,38)=1.23, MSE=0.03 , p=.274 , $\eta_{p}^{2}=.03$ ), but were higher in both collaborative conditions relative to the control condition ( Fs(1,38)≥38.80 , ps<.001 , $\eta_{p}^{2}\geq .45$ ).

A further 2 × 3 ANOVA on correct identifications of cooperators and cheaters only showed a significant main effect of group condition ( F(2,57)=15.87 , MSE=0.04 , p<.001 , $\eta_{p}^{2}=.36$ ), but no further significant effects ( Fs≤0.19 , ps≥.667 , $\eta_{p}^{2}\leq .003$ ). Similar to hit rates, correct identifications did not differ between the two collaborative conditions ( F(1,38)=0.68 , MSE=0.04 , p=.414 , $\eta_{p}^{2}=.02$ ), but were enhanced in both collaborative conditions relative to the control condition ( Fs(1,38)≥21.44 , ps<.001 , $\eta_{p}^{2}\geq .36$ ).^3^

### Discussion

Changing the interpolated memory test and merging old/new judgment and player identification into one step in Experiment 3 did not seem to have the desired effect. Participants in the regular collaboration condition were not better able to use socially shared information to improve their subsequent decisions. Indeed, relative to Experiment 2, the main difference in Experiment 3 was that participants in the nominal control condition were more likely to cooperate with any new type of player (distractor players as well as cheaters and cooperators that were encountered by other group members, which they could not know about). Potentially, the merged judgment may have made it more difficult for participants to clearly differentiate between old and new players, especially when there was no interaction with other group members who could have corrected them.

Adding a collaboration condition with inclusive instructions to Experiment 3 did also not enhance the amount of information that was socially shared during the interpolated memory test relative to the regular collaboration condition. Moreover, the two collaboration conditions did not differ in terms of cooperation behavior on the final decision game. Even with inclusive instructions, cooperation rates were not enhanced relative to the control condition without collaboration. Participants were generally a bit more likely to cooperate with cooperators, who had previously only cooperated with other group members. Although, descriptively, this numerical pattern seemed most pronounced in the collaboration condition with inclusive instructions, the critical interaction effect was not significant. Moreover, additional Bayesian analyses suggest caution when interpreting the overall finding, because the evidence for a main effect of player type was inconclusive. In sum, a switch in collaboration instructions in order to motivate participants to try to benefit from collaboration did also not seem to have the desired effect.

### Additional analysis on the pooled data from Experiments 1 to 3: overlap of individual decisions at the group level

Several studies on collaborative remembering suggest that collaboration not only benefits individual memory, but also synchronizes the specific contents remembered by group members (i.e., after collaboration, group members tend to remember a higher proportion of identical contents). Whether collaborative remembering also prompts a synchronization of subsequent behavior that is based upon the remembered contents has not been examined to date. Although this was not the primary goal of the present study, the pooled data across Experiments 1 to 3 can be used to address the issue. Individuals’ decision behavior may not have become vastly more adaptive as a consequence of prior collaboration, but potentially, it might still have become more similar.

The analysis compared the proportion of identical decisions made by participants from collaborative groups (without specific instructions) to those made by participants from control conditions without collaboration. To keep number of groups across conditions constant, the additional collaborative condition with inclusive instructions in Experiment 3 was not included in this analysis. The group level analysis thus rested on a total of 50 collaborative and 50 non-collaborative triads (with 15 triads per condition from Experiments 1 and 2, respectively, and another 20 triads from Experiment 3).

A first 2 × 2 ANOVA with the two factors of group condition (collaborative, nominal) and player type (cooperator, cheater) was run to examine proportions of all identical decisions that were made uniformly by all three members of a group. The ANOVA showed a significant main effect of group condition ( F(1,98)=9.34 , MSE=0.02 , p=.003, $\eta_{p}^{2}=.09$ ) and a significant main effect of player type ( F(1,98)=10.35 , MSE=0.02 , p=.002 , $\eta_{p}^{2}=.09$ ). An interpolated collaborative memory test generally enhanced the proportion of identical decisions that were subsequently made by individual group members (0.36 vs. 0.30); moreover, the overlap was higher when the decisions involved cheaters rather than collaborators (0.36 vs. 0.30). The interaction effect was not significant ( F(1,98)=0.37 , MSE=0.02 , p=.545 , $\eta_{p}^{2}=.004$ ).

**Figure 7.**
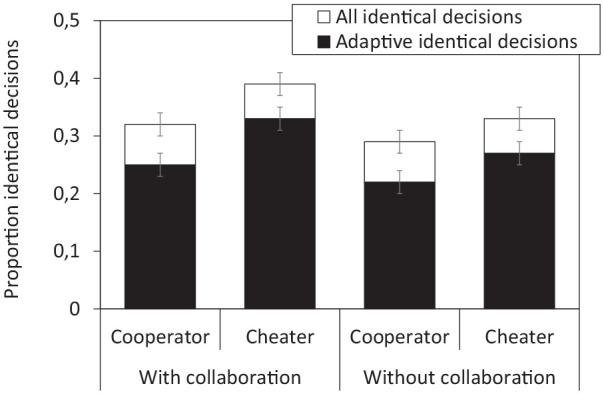
Mean proportion of identical decisions across all three group members, based on the pooled data from Experiments 1 to 3. The stacked bars show all identical decisions; the black bars show adaptive identical decisions only (i.e., cooperation with cooperators, no cooperation with cheaters). Error bars represent ±1 standard error of the mean.

A second 2 × 2 ANOVA examined proportions of adaptive decisions uniformly made by all group members (i.e., decisions to cooperate with cooperators, or not to cooperate with cheaters). The pattern was similar to the one reported above, with significant main effects of group condition ( F(1,98)=5.34 , MSE=0.02 , p=.023 , $\eta_{p}^{2}=.05$ ) and player type ( F(1,98)=9.92 , MSE=0.02 , p=.002 , $\eta_{p}^{2}=.09$ ), but no significant interaction ( F(1,98)=0.41 , MSE=0.02 , p=.523 , $\eta_{p}^{2}=.004$ ). Collaboration enhanced the overlap in adaptive decisions (0.29 vs. 0.24); moreover, the overlap was again higher for cheaters rather than collaborators (0.30 vs. 0.24; see also Figure 7).

In sum, these additional analyses on the pooled data of Experiments 1 to 3 suggest that prior collaborative remembering may have prompted greater overlap in later individual decisions at the group level—even though it did not have a particular benefit at the individual level. Greater overlap after collaboration was observed irrespective of whether only adaptive or all decisions were analyzed, and in both cases player type (cooperator, cheater) had an additional influence.

## General discussion

The present study set out to explore to what degree effects of collaborative remembering could serve a directive function and guide future behavior. Three experiments, however, found hardly any evidence for a directive function of collaborative remembering in the context of a subsequent decision task. In all experiments, participants generally showed adaptive decision behavior and chose to cooperate more with previously encountered cooperators than cheaters or new players, thus replicating prior work (see Schaper et al., 2019). This adaptive behavior was, however, not systematically enhanced above baseline performance on the basis of interpolated collaborative remembering—neither for initially studied information nor for information that was studied by other group members only.

### No directive function of collaborative remembering?

On the one hand, these results might suggest that effects of collaboration on individual memory do not easily carry over to subsequent tasks that rely on socially shared information. Numerous studies have shown effects of collaborative remembering on subsequent individual memory, and importantly, these effects also seem to arise with synchronous online testing as was also used in the present study (Abel & Bäuml, 2023; see also Conte et al., 2023; Rossi-Arnaud et al., 2023). Moreover, effects of collaborative remembering have not only been observed with simple words as stimulus materials, but also with more complex ones. For example, collaborative facilitation has been shown with emotional pictures (Choi et al., 2017), videos (Bärthel et al., 2017; Ekeocha & Brennan, 2008), or stories (Weldon & Bellinger, 1997) as study materials, and it may be present in both item and source memory (Nie et al., 2023). Social contagion is in fact often examined with more complex information, like pictures of household scenes (Hart & Meade, 2021; Roediger et al., 2001) or materials showing crimes (Echterhoff et al., 2005; French et al., 2008; Gabbert et al., 2003). Yet, even if participants’ memory is influenced by collaboration, this influence may not necessarily be strong enough to have cascading effects in subsequent situations. Put differently, social information transmission may not necessarily help to direct subsequent individual behavior in a decisive manner. This holds while there may of course be different scenarios, in which social input may be more likely to affect an individual’s behavior (e.g., when an individual seeks out and receives another person’s targeted advice on an imminent problem).

Indeed, the present null finding on effects of collaborative remembering on subsequent decision making seem to be in good company. Although there is some evidence that collaboration can benefit strategy selection in specific problem-solving tasks (e.g., Laughlin et al., 2008), the case seems to be different when problem solving or decision making depend upon previously encoded information. Indeed, a separate body of work seems to suggest that group discussions do little to improve decision making in groups. Interestingly, here, the lack of a beneficial effect has been attributed to the fact that unrestricted group discussions often tend to focus on information that is already known and shared by everyone—and neglect unshared information that is only known by single group members (Stasser & Titus, 1985, 1987; see also Reimer et al., 2010; Stasser & Abele, 2020; Wittenbaum & Park, 2001). In contrast, the present study used a collaborative recognition task, in which all shared and unshared pieces of information are discussed by default during the collaboration phase. Thus, the null findings in the present study could suggest that the lack of a beneficial effect of groups on decision making may not just hinge on groups being selective in the contents of their discussions; even when all contents are discussed, benefits do not necessarily ensue.

A study by Bell et al. (2014) examined memory for reputational information in individuals. The results of this study suggested that episodes that involved cheating were particularly well remembered when they were linked to personal costs for the participants. Potentially, personal consequences may be critical for the link between memory and subsequent decision making. Although it can be adaptive to incorporate other people’s experiences into one’s own representation of the world, hearing about personal consequences suffered by another person may not have the same immediate relevance and may, therefore, leave less of an impression in an individual’s mind. More work is needed to directly address this suggestion. In this regard, a limitation of the present study is that it cannot address with certainty whether collaborative remembering did in fact influence individuals’ memory for cooperators and cheaters that they or other group members had previously encountered. The experiments did incorporate a final individual memory test, but this test was always completed at the end of each experiment and is thus contaminated by prior completion of the final decision game (for a brief summary, see the OSF project page).

### The potential influence of task features

On the other hand, it is also important to note that the null effects in the present experiments could be connected to the specific decision task that was used. The task was chosen because prior work had demonstrated a link between individual memory and adaptive decision making in this task (Schaper et al., 2019), and the prediction was that enhanced individual memory on the basis of collaboration should further increase adaptive decision making. The results were not consistent with this prediction, but task features may have potentially influenced the results.

In particular, collaboration in the present experiments may have made participants skeptical to rely on other group members’ input. One aspect to consider is that the decision task relies on the differentiation of cooperators and cheaters, which in itself might make participants cautious to trust others. A further aspect may, however, also be information load. Although the proportion of studied to unstudied information in the present experiments was similar to prior work (Abel & Bäuml, 2020, 2023), a lower number of trials and information units was used, due to the concern that faces and associated behaviors might be more difficult to memorize than relatively simple word materials. As a consequence, it may have been easier for participants to unequivocally determine whether or not they themselves had encountered specific players before. Indeed, the results suggest that participants were quite conservative in their later decision making, for the most part handling players that had been encountered by other group members the same as completely new players that had not been encountered by anyone.

In future work, the replicability of the present null findings should be examined in different decision tasks, which do not rely on differentiation of cooperators and cheaters. Apart from considering the potential need for a greater amount of to-be-studied information, it should also be considered how observed decision behavior can be interpreted. An additional analysis across the pooled data of all experiments suggested that collaboration had a small synchronizing effect, such that individuals from collaborative groups were slightly more likely to make identical decisions later. Although this could be seen as consistent with a social function of collaborative remembering, this greater overlap may, however, have arisen for different reasons across group members. For example, for a particular cheater, some group members may have decided not to cooperate because they had no memory of the player, whereas others were in fact able to recollect that the player was a cheater. Even though collaboration prompted a small increase in overlapping behavior, the basis of this overlap is a bit unclear. In future work, simpler tasks might allow for clearer conclusions.

## Summary

The present experiments found no influence of collaborative remembering on subsequent decision making in a modified version of a prisoner’s dilemma. All participants showed adaptive decision making in this task; this pattern was, however, not further improved by an opportunity for social information transmission. Prior work has suggested that collaborative facilitation and social contagion may serve a social function by supporting the development of shared memories (Abel & Bäuml, 2023; see also Choi et al., 2017). In contrast, the present findings seem to suggest that effects of collaborative remembering do not necessarily serve a directive function for individuals, although it is important to note that task characteristics could have played a role. Future work is needed to confirm these findings in different decision tasks.

## Notes

1. The focus of the experiment was on individual decisions in the final decision game, and the main analyses were run on these data. Performance on the final source memory test was analyzed as well; for brevity, and because performance on this test is likely contaminated by prior completion of the decision game, the data are, however, not reported in this study, but can be found on the OSF project page (https://osf.io/mcj7k/).

2. Correct player identifications on the interpolated test were analyzed as proportions of correct answers, because the goal was to (roughly) address whether a sufficient amount of information was socially shared in collaborative groups. Had the goal been to address source memory performance, different approaches would have been more useful (like assessing conditional source memory performance, relative to hit rates; or using cognitive modeling to more clearly distinguish between item memory, source memory, and guessing biases; e.g., Bayen et al., 1996, Bröder & Meiser, 2007).

3. A general concern regarding the analyses on interpolated test performance could be that conclusions might differ depending on the random subsample that is drawn from the individual control condition. To address this concern, the individual control condition in each experiment was split up into three random subsamples, and all analyses were repeated to evaluate whether different conclusions would be drawn depending on what subsample was used. For all three experiments, the main conclusions remained the same, however, irrespective of which subsamples were used for the analyses.
